# Machine learning pre-hospital real-time cardiac arrest outcome prediction (PReCAP) using time-adaptive cohort model based on the Pan-Asian Resuscitation Outcome Study

**DOI:** 10.1038/s41598-023-45767-z

**Published:** 2023-11-21

**Authors:** Hansol Chang, Ji Woong Kim, Weon Jung, Sejin Heo, Se Uk Lee, Taerim Kim, Sung Yeon Hwang, Sang Do Shin, Won Chul Cha, Pan-Asian Resuscitation Outcomes Study Clinical Research Network investigators, Marcus Ong

**Affiliations:** 1 Department of Emergency Medicine, Samsung Medical Center, Sungkyunkwan University School of Medicine, 115 Irwon-ro Gangnam-gu, Seoul, 06355 Republic of Korea; 2 Department of Digital Health, Samsung Advanced Institute for Health Science & Technology (SAIHST), Sungkyunkwan University, 115 Irwon-ro Gangnam-gu, Seoul, 06355 Republic of Korea; 3 LG UPLUS, 71, Magokjungang 8-ro, Gangseo-gu, Seoul, 07795 Republic of Korea; 4 Smart Health Lab, Research Institute of Future Medicine, Samsung Medical Center, 81 Irwon-ro Gangnam-gu, Seoul, 06351 Korea; 5 Department of Emergency Medicine, Seoul National University Hospital, Seoul, Korea; 6 Digital Innovation, Samsung Medical Center, 81 Irwon-ro Gangnam-gu, Seoul, 06351 Republic of Korea; 7 Department of Emergency Medicine, Samsung Medical Center, Sungkyunkwan University School of Medicine, 81 Irwon-ro, Gangnam-gu, Seoul, 06351 Republic of Korea; 8 Department of Emergency Medicine, Singapore General Hospital, Outram Road, Singapore, 169608 Singapore

**Keywords:** Medical research, Prognosis

## Abstract

To save time during transport, where resuscitation quality can degrade in a moving ambulance, it would be prudent to continue the resuscitation on scene if there is a high likelihood of ROSC occurring at the scene. We developed the pre-hospital real-time cardiac arrest outcome prediction (PReCAP) model to predict ROSC at the scene using prehospital input variables with time-adaptive cohort. The patient survival at discharge from the emergency department (ED), the 30-day survival rate, and the final Cerebral Performance Category (CPC) were secondary prediction outcomes in this study. The Pan-Asian Resuscitation Outcome Study (PAROS) database, which includes out-of-hospital cardiac arrest (OHCA) patients transferred by emergency medical service in Asia between 2009 and 2018, was utilized for this study. From the variables available in the PAROS database, we selected relevant variables to predict OHCA outcomes. Light gradient-boosting machine (LightGBM) was used to build the PReCAP model. Between 2009 and 2018, 157,654 patients in the PAROS database were enrolled in our study. In terms of prediction of ROSC on scene, the PReCAP had an AUROC score between 0.85 and 0.87. The PReCAP had an AUROC score between 0.91 and 0.93 for predicting survived to discharge from ED, and an AUROC score between 0.80 and 0.86 for predicting the 30-day survival. The PReCAP predicted CPC with an AUROC score ranging from 0.84 to 0.91. The feature importance differed with time in the PReCAP model prediction of ROSC on scene. Using the PAROS database**,** PReCAP predicted ROSC on scene, survival to discharge from ED, 30-day survival, and CPC for each minute with an AUROC score ranging from 0.8 to 0.93. As this model used a multi-national database, it might be applicable for a variety of environments and populations.

## Introduction

Prehospital care plays an essential role in the management of out-of-hospital cardiac arrest (OHCA)^1–3^. Previous studies have identified several components of prehospital management that is highly associated with OHCA patient outcome, including chest compression time, defibrillation, and transport^3–6^. However, it is still challenging to make clinical decisions in OHCA settings due to the difficulties in identifying the cause and estimating the patient's resuscitation prognosis.

It is difficult to predict whether return of spontaneous circulation (ROSC) can be achieved on the scene without providing advanced cardiac life support in a hospital, which makes it unclear when to perform “scoop and run” to the hospital for advanced cardiac life support (ACLS)^7–9^. To save time during transport, where resuscitation quality can degrade in a moving ambulance, it would be prudent to perform “stay and play” resuscitation if there is a high likelihood of ROSC occurring at the scene^10–13^. However, a hospital would obviously apply more resources and provide optimal management when ROSC is difficult due to unknown medical causes^7,9^. It makes it more difficult to make decisions during the prehospital phase because, although it is time-sensitive and influenced by the scene environment, less information and resources are available than during the in-hospital phase^14,15^.

Prior to this study, multiple publications suggested a machine learning-based prediction model of OHCA patient outcomes or analyzed the relevance of each predictor with ROSC^16–19^. Some models were built to be used in the prehospital phase for predicting on-scene ROSC^20,21^. The situation at the scene might keep varying, and is especially time-dependent in the prehospital stage of OHCA^15,22^. A real-time prediction model might therefore be beneficial for emergency medical service (EMS) prehospital resuscitation.

In this study, we aimed to develop a pre-hospital real-time cardiac arrest outcome prediction model using time-adaptive cohort to provide real-time prediction of ROSC on scene and further prognosis, during the EMS resuscitation attempt.

## Methods

This study was approved by the Institutional Review Board (IRB) of the Samsung Medical Center (IRB No. 2022-04-093). The need for informed consent was waived by of the Samsung Medical Center IRB because of the retrospective, observational, and anonymous nature of the study. All the methods were performed in accordance with relevant guidelines and regulations. Because it was inappropriate, it was not possible to involve patients or the public in the design, conduct, reporting, or dissemination plans of our research.

We developed the pre-hospital real-time cardiac arrest outcome prediction (PReCAP) model to predict the ROSC on screen and other patient outcomes using prehospital input variables with time-adaptive cohort.

### Study setting and population

The Pan-Asian Resuscitation Outcome Study (PAROS) database, which included OHCA patients transferred by emergency medical service (EMS) in Asia between 2009 and 2018, was used for this study. Seven Pacific Asia nations were included. Patients in the PAROS database who were under the age of 18 were excluded. Patients who were not transported by EMS transport but with private or public transport were also excluded. Patients without an EMS team resuscitation attempt or patients whose arrest occurred after hospital arrival were excluded. Finally, patients who were missing at least one of the essential time information, including the date and time of the arrest incidence and the time of call received at the dispatch center were excluded.

### Primary outcome

This research aimed to predict ROSC at the scene. ROSC at the scene refers to ROSC during the prehospital phase, prior to hospital arrival Scene ROSC information was recorded by each country’s EMS and collected from the PAROS database.

### Secondary outcome

The patient survival to discharge from the emergency department (ED), the 30-day survival rate, and the final Cerebral Performance Category (CPC) were secondary prediction outcomes in this study. Survival to discharge from the ED refers to whether a patient is admitted, transferred, or discharged for further care in a state of survival. Thirty days of survival refers to whether the patient survived 30 days following cardiac arrest. Whether the patient's final CPC was 1 or 2 was also predicted as an outcome.

### Selection of predictor and preprocessing

**Figure 1:**
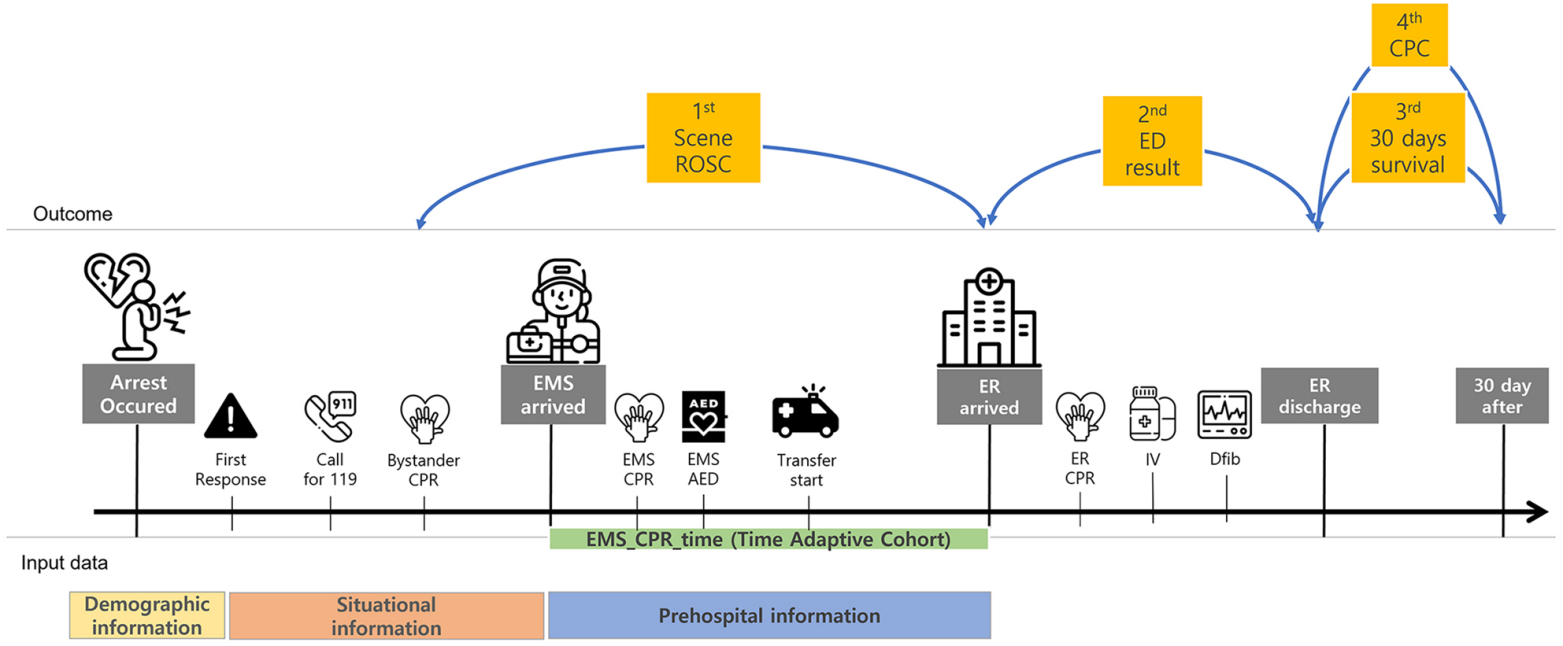
Overall study flow in the development of PReCAP (Pre-hospital Real-time Cardiac Arrest outcome Prediction model). CPR, cardiopulmonary resuscitation; EMS, emergency medical service; AED, automated external defibrillator; ER, emergency room; ED, emergency department; Dfib, defibrillation.

From variables available in the PAROS database, we selected relevant variables to predict OHCA outcomes. Demographic information, including city, sex, race, and age was collected. Scene environment information, including location type and time, was included. Location type was divided into home residence, health care facility, public building, nursing home, street, industrial area, transport center, place of recreation, ambulance, and others. Prehospital cardiopulmonary resuscitation (CPR) information, including information about witnessed arrest, bystander CPR information, first responder, information about automated external defibrillator (AED) usage by bystander or EMS, first rhythm of patient, prehospital airway method, and prehospital epinephrine administration, was also included (Fig. 1, Table S1 of Multimedia Appendix 1).

### Handling missing data

Patients with missing essential time information, such as time of arrival of the ambulance at the scene and its arrival at the ED were excluded from the study. Missing information about categorical variables were considered as ‘unknown.’ Missing information about numeric variables was not included in the statical analysis. In outcome variable, unknown outcome was considered as negative outcome. Variables with more than 70% of missing values were excluded in model development.

### Time definition and time-adaptive cohort development

For real-time prediction, we considered developing time-adaptive cohorts and created prediction models for each time cohort. By dividing the total prehospital CPR time by one minute, a time-adaptive cohort was developed. Total prehospital CPR time was defined as the amount of time between the arrival of the ambulance at the scene and its arrival at the ED. Each minute of ongoing CPR from the time the ambulance arrived at the scene is defined as an adaptive cohort. The PReCAP model for each minute was developed based on each cohort. The time-adaptive cohort was developed by each minute; the training set and test set were divided separately in each cohort. Therefore, the PReCAP prediction model for every minute was also developed independently.

### Model development

We used time-adaptive cohort to develop a pre-hospital real-time prediction model, PReCAP. We trained PReCAP with each data set for every minute.The PReCAP consisted of multiple models that were trained with multiple data sets throughout time. CPR duration from the scene to the hospital was not included as a machine learning model’s training feature but used as a dividing criterion of the time-adaptive cohort.

We compare the performances of PreCAP with the non-time adaptive model. The non-time adaptive model would be the same model with the 0 min point of the CPR patient cohort, which includes all patients in PAROS, because the non-time adaptive model refers to a model that predicts scene ROSC and other outcomes based on all patient cohorts with the same variables as PReCAP without using time-adaptive cohorts.

To develop PReCAP, we additionally developed three models, random forest, light gradient-boosting machine (LightGBM), and extreme gradient boosting (XGBoost) as a machine learning algorithm. The implemented software for model development included the Python programming language (version 3.8.5), the Tensorflow framework (version 2.3.1), and scikit-learn (version 0.23.2). For model development, LightGBM, which is a Python package, was used. LightGBM uses a leaf-wise method, which is different from other level-wise methods of tree-based algorithms. Therefore, LightGBM shows faster and better performance in the tuble structured dataset^23^. There are various algorithms for training machine learning models, but the tree-based ensemble model shows the best performance for classification of structured data. We compared the area under the receiver operating characteristic curve (AUROC) to find the best performance and chose LightGBM as a PReCAP algorithm.

### Statistical analysis

In the descriptive analysis of variables of interest, variables were summarized by frequency and percentage. Comparisons between groups (ROSC vs. non-ROSC) were performed using χ2 test at a 0.05 significance level. Multivariable analysis was performed to show odds ratio of input features to ROSC on scene. All statistical analyses were performed using R (version 4.1.1; R Foundation for Statistical Computing, Vienna, Austria).

## Results

### Population and demographic information

**Figure 2:**
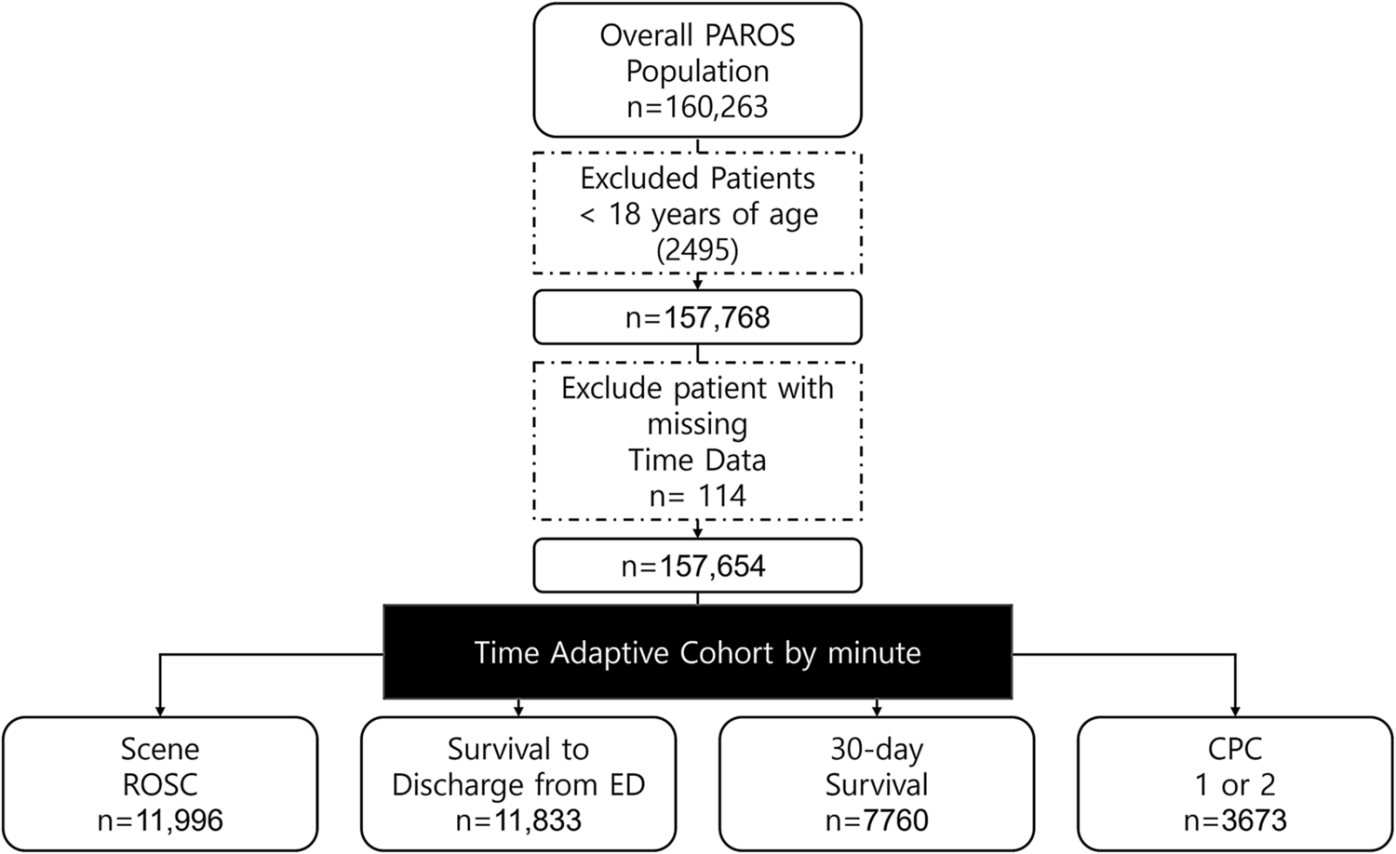
Study population.

Between 2009 and 2018, the PAROS database had 160,263 OHCA patients from 13 Asian nations (Table S1 of Multimedia Appendix 1). Patients under the age of 18 (2495) and patients without proper time data (114) were excluded from time-adaptive cohort configuration. Finally, 157,654 patients were enrolled in our study (Fig. 2 of Multimedia Appendix 1).

The basic characteristics of the variables considered in building the model is shown in Table 1. Out of a total population of 160,263 patients, ROSC on scene was accomplished in 11,995 individuals.

**Table 1:** Patient characteristics.

	ROSC at scene/enroute		*P* value
	No (N = 145,656)	Yes (N = 11,995)	
Location type			< 0.001
Home residence	35,687 (24.5%)	2160 (18%)	
Healthcare facility	685 (0.5%)	99 (0.8%)	
Public/Commercial building	3112 (2.1%)	473 (3.9%)	
Nursing home	3519 (2.4%)	210 (1.8%)	
Street/highway	3375 (2.3%)	290 (2.4%)	
Industrial area	757 (0.5%)	66 (0.6%)	
Transport center	203 (0.1%)	28 (0.2%)	
Place of recreation	654 (0.4%)	107 (0.9%)	
In EMS/Private ambulance	93 (0.1%)	15 (0.1%)	
Other	2936 (2%)	255 (2.1%)	
Unknown	94,635 (65%)	8292 (69.1%)	
Gender			< 0.001
Female	60,354 (41.4%)	4120 (34.3%)	
Male	85,302 (58.6%)	7875 (65.7%)	
Age			< 0.001
< 60	31,086 (21.3%)	3064 (25.5%)	
60–69	22,371 (15.4%)	2340 (19.5%)	
70–79	34,349 (23.6%)	2932 (24.4%)	
> = 80	57,850 (39.7%)	3659 (30.5%)	
Cause of arrest			0.001
Trauma	593 (0.4%)	23 (0.2%)	
Non-trauma	4752 (3.3%)	388 (3.2%)	
Unknown	140,311 (96.3%)	11,584 (96.6%)	
Arrest witnessed by			< 0.001
Not witnessed	88,633 (60.9%)	3134 (26.1%)	
EMS/Private ambulance	823 (0.6%)	134 (1.1%)	
Bystander-healthcare provider	52,813 (36.3%)	8590 (71.6%)	
Unknown	3387 (2.3%)	137 (1.1%)	
Bystander CPR			< 0.001
Yes	62,501 (42.9%)	6302 (52.5%)	
No	82,935 (56.9%)	5682 (47.4%)	
Unknown	220 (0.2%)	11 (0.1%)	
First CPR initiated by			< 0.001
No CPR initiated	9499 (6.5%)	637 (5.3%)	
First responder	571 (0.4%)	108 (0.9%)	
Ambulance crew	26,803 (18.4%)	1513 (12.6%)	
Bystander	11,398 (7.8%)	1794 (15%)	
Unknown	97,385 (66.9%)	7943 (66.2%)	
Bystander applied AED			< 0.001
Yes	1513 (1%)	338 (2.8%)	
No	123,423 (84.7%)	9881 (82.4%)	
Unknown	20,720 (14.2%)	1776 (14.8%)	
First arrest rhythm			< 0.001
VF	8532 (5.9%)	3226 (26.9%)	
VT	313 (0.2%)	106 (0.9%)	
PEA	23,419 (16.1%)	3917 (32.7%)	
Asystole	94,177 (64.7%)	2627 (21.9%)	
Unknown	19,215 (13.2%)	2119 (17.7%)	
Prehospital defibrillation			< 0.001
Yes	18,412 (12.6%)	4588 (38.2%)	
No	127,239 (87.4%)	7407 (61.8%)	
Unknown	5 (0%)	0 (0%)	
Defibrillation performed by first responder			< 0.001
Yes	573 (0.4%)	239 (2%)	
No	103,729 (71.2%)	8083 (67.4%)	
Unknown	41,354 (28.4%)	3673 (30.6%)	
Defibrillation performed by ambulance crew			< 0.001
Yes	11,713 (8%)	2920 (24.3%)	
No	116,652 (80.1%)	7184 (59.9%)	
Unknown	17,291 (11.9%)	1891 (15.8%)	
Defibrillation performed by bystander-healthcare provider			< 0.001
Yes	81 (0.1%)	27 (0.2%)	
No	12,145 (8.3%)	858 (7.2%)	
Unknown	133,430 (91.6%)	11,110 (92.6%)	
Defibrillation performed by bystander-lay person			< 0.001
Yes	142 (0.1%)	50 (0.4%)	
No	104,161 (71.5%)	8285 (69.1%)	
Unknown	41,353 (28.4%)	3660 (30.5%)	
Defibrillation performed by bystander-family			< 0.001
Yes	108 (0.1%)	17 (0.1%)	
No	12,145 (8.3%)	858 (7.2%)	
Unknown	133,403 (91.6%)	11,120 (92.7%)	
Mechanical CPR device used by EMS/private ambulance			< 0.001
Yes	6798 (4.7%)	490 (4.1%)	
No	69,018 (47.4%)	5124 (42.7%)	
Unknown	69,840 (47.9%)	6381 (53.2%)	
Prehospital advanced airway			< 0.001
Yes	59,473 (40.8%)	5410 (45.1%)	
No	85,096 (58.4%)	6546 (54.6%)	
Unknown	1087 (0.7%)	39 (0.3%)	
Type of advanced airway method			< 0.001
No advanced airway	85,096 (58.4%)	6546 (54.6%)	
Oral/nasal ET	10,059 (6.9%)	1449 (12.1%)	
Combitube	69 (0%)	7 (0.1%)	
LMA	19,593 (13.5%)	1322 (11%)	
King airway	21,587 (14.8%)	2084 (17.4%)	
Other	8163 (5.6%)	548 (4.6%)	
Unknown	1089 (0.7%)	39 (0.3%)	
Prehospital epinephrine administered			< 0.001
Yes	15,794 (10.8%)	4420 (36.8%)	
No	128,482 (88.2%)	7513 (62.6%)	
Unknown	1380 (0.9%)	62 (0.5%)	

ROSC, return of spontaneous circulation; EMS, emergency medical service; CPR, cardiopulmonary resuscitation; AED, automated external defibrillator; VT, ventricular tachycardia; VF, ventricular fibrillation; PEA, pulseless electrical activity; ET, endotracheal intubation.

**Figure 3:**
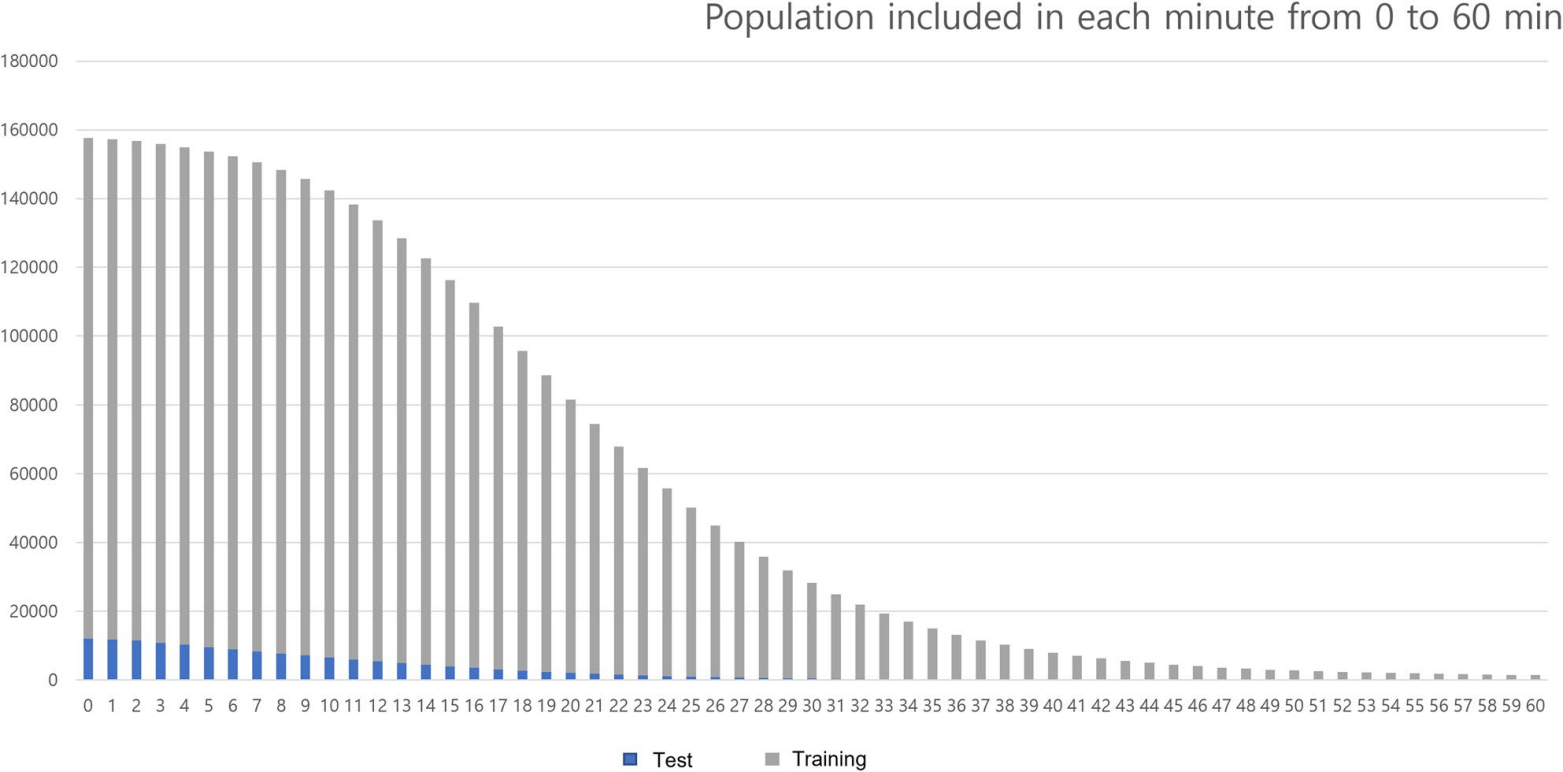
Population included for each minute from 0 to 60 min for developing time-adaptive cohorts. The PReCAP model was built independently for each minute, with an independent time cohort.

Time-adaptive cohorts were created for each minute of CPR performed at the scene. Each time-adaptive cohort was randomly split into training and test sets (Fig. 3 of Multimedia Appendix 1).

### Prediction outcome

Table 2 displays the major outcome of PReCAP at the time of arrival of EMS team in scene, 5 min, and 10 min following the EMS team’s arrival at the scene of the arrest. Table 2 presents the PReCAP prediction results for each outcome. In terms of prediction of ROSC on scene, the PReCAP had an AUROC score between 0.85 and 0.87 (Table S3 of Multimedia Appendix 1). The PReCAP had an AUROC score between 0.91 and 0.93 for predicting survived to discharge from ED (Table S4 of Multimedia Appendix 1), and an AUROC score between 0.80 and 0.86 for predicting the 30-day survival (Table S5 of Multimedia Appendix 1). The PReCAP predicted CPC with an AUROC score ranging from 0.84 to 0.91 (Table S6 of Multimedia Appendix 1).

**Table 2:** PReCAP (Pre-hospital Real-time Cardiac Arrest outcome Prediction) model AUROC by outcome.

	Scene ROSC	Emergency Department survival discharge	30-day survival	CPC 1 or 2
0 min^a^	0.864	0.918	0.852	0.917
1 min	0.864	0.919	0.851	0.915
2 min	0.864	0.918	0.848	0.915
3 min	0.863	0.918	0.846	0.914
4 min	0.862	0.917	0.842	0.911
5 min	0.862	0.916	0.840	0.908
6 min	0.861	0.916	0.835	0.902
7 min	0.860	0.917	0.833	0.902
8 min	0.862	0.918	0.829	0.899
9 min	0.862	0.918	0.824	0.892
10 min	0.866	0.920	0.821	0.887
11 min	0.867	0.921	0.822	0.882
12 min	0.868	0.923	0.824	0.871
13 min	0.870	0.924	0.821	0.871
14 min	0.867	0.924	0.817	0.869
15 min	0.872	0.926	0.819	0.864
16 min	0.870	0.929	0.811	0.866
17 min	0.868	0.929	0.807	0.870
18 min	0.866	0.930	0.802	0.864
19 min	0.868	0.931	0.805	0.835
20 min	0.867	0.932	0.803	0.840

AUROC, area under receiver operating curve; CPC, Cerebral Performance Category.

^a^Same as non-time adaptivemodel, which predicts outcome without time-adaptive cohort.

**Figure 4:**
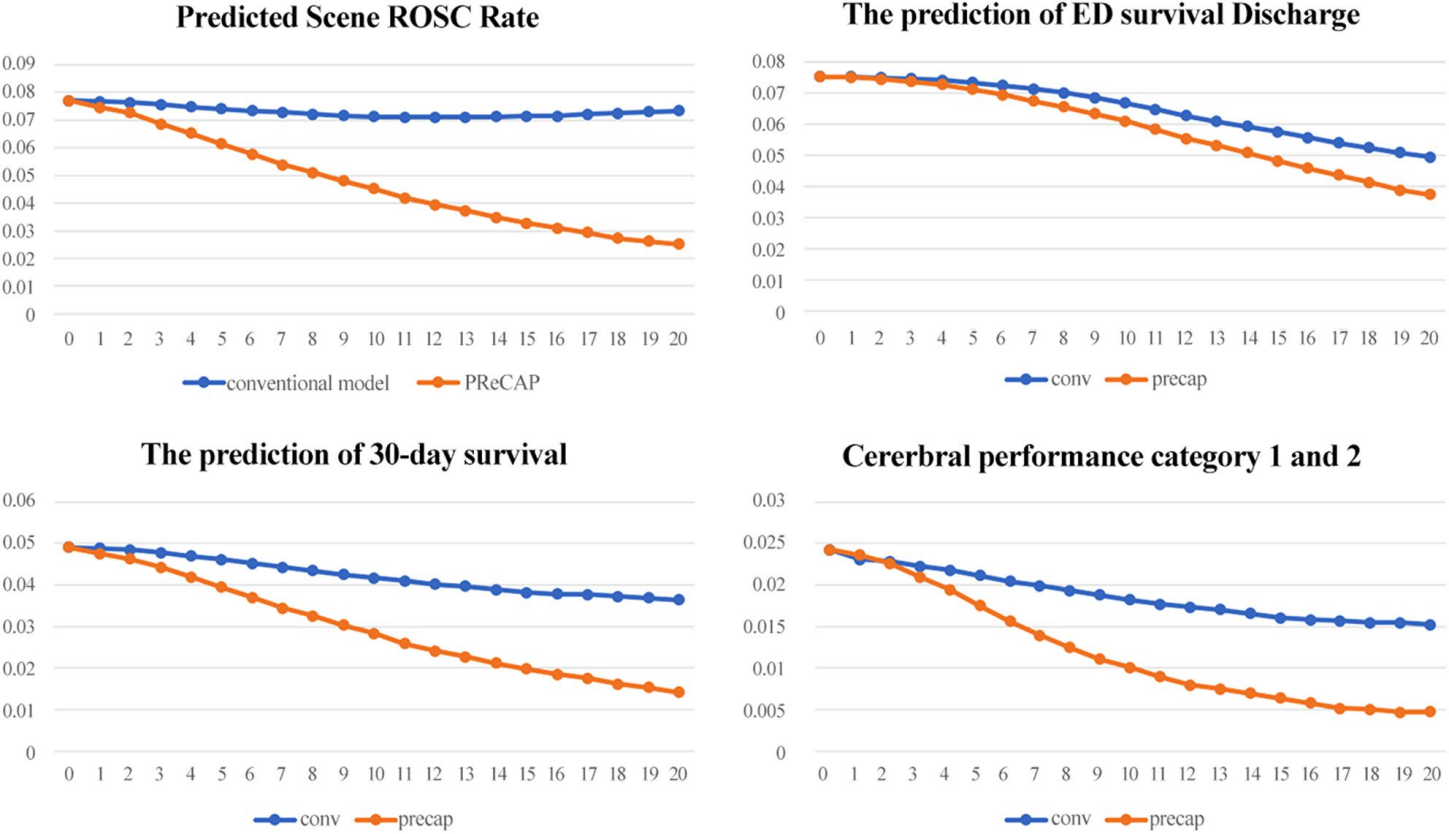
Predicted scene return of spontaneous circulation ROSC, survival to discharge from the emergency department (ED), the 30-day survival rate, and the final Cerebral Performance Category (CPC). The prediction rate of non-time adaptive model is shown as a blue line while the prediction rate of PReCAP is shown as an orange line.

Figure 4 shows the predicted survival rate by time in the PReCAP model and conventional model, which predicts ROSC on scene without using time-adaptive cohort. PReCAP model shows lower predicted survival rate than conventional model as time goes on (Fig. 2, Table S7 of Multimedia Appendix 1).

### Feature analysis

**Figure 5:**
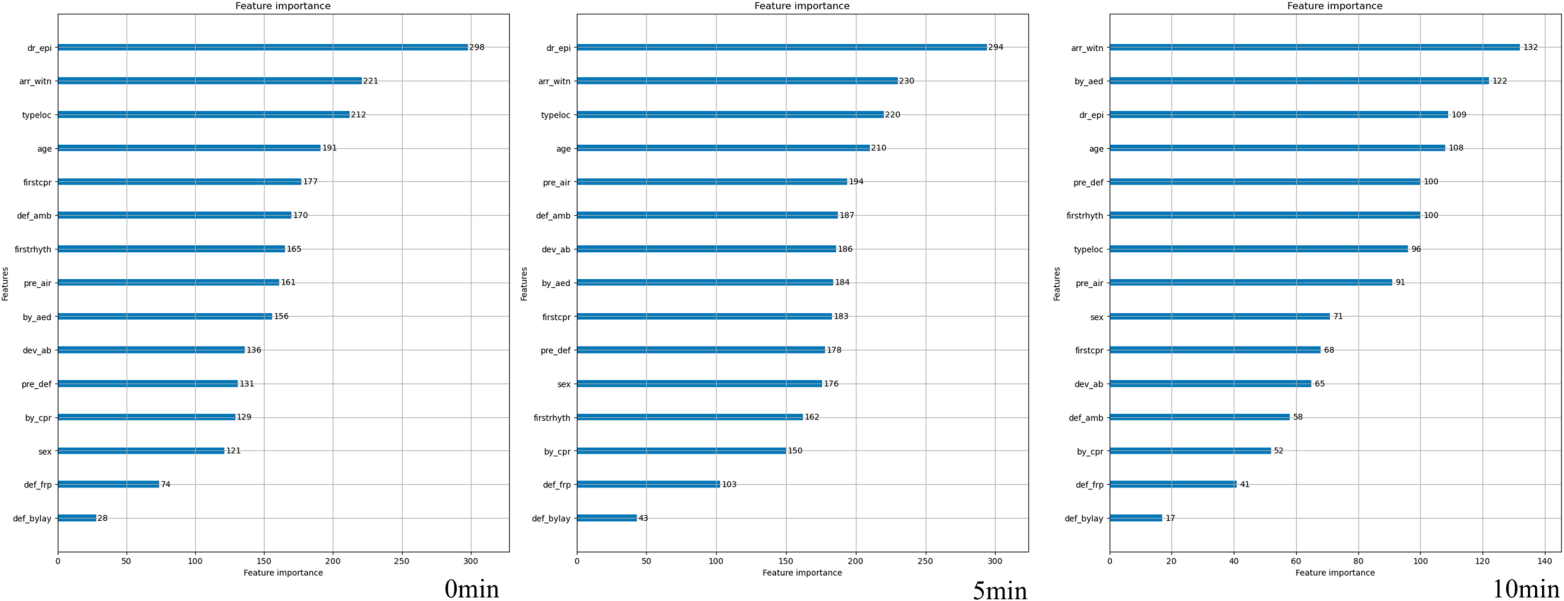
Feature importance of ROSC on scene in Pre-Hospital Real Time Precision Model (PReCAP) at 0 min, 5 min and 10 min**.** noflow_time, Estimated Occurrence time to response time; bystander_time, Bystander CPR time; dr_epi, Prehospital Epinephrine injection; firstrhyth, First Rhythm; typeloc, Location type; pre_air, Prehospital advanced airway; arr_witn, witnessed arrest; by_aed, Bystander AED done; def_amb, Defibrillation in ambulance; frstcpr, first CPR initiator; by_cpr, Bystander CPR done; dev_ab, mechanical CPR device used by EMS/private ambulance; pre_def, prehospital defibrillation; def_frp, defibrilation performed by first responder; def_bylay, defibrillation performed by bystander-lay person.

Figure 5 and Figs. S1–S3 of Multimedia Appendix 1 illustrate the feature importance of ROSC on scene in PReCAP prediction at 0 min, 5 min, and 10 min after EMS CPR. Age, ambulance arrival time, and initial rhythm were the most influential factors in each time cohort. However, the importance of features changed throughout time. In the early 0 min PReCAP model, for instance, the administration of epinephrine was the fourth important feature. After 5 min, in the PReCAP ROSC on scene prediction, the fourth critical aspect was the prehospital airway. People who observed the arrest after 10 min constituted the fourth important feature. As the prediction model was constructed every minute, the value of importance of certain features likewise evolved over time.

In addition, a multivariate analysis was conducted to determine the relationship between ROSC on scene and each feature (Table 3). In a multivariate analysis, the factors with significant differences in the univariate analysis were analyzed.

**Table 3:** Multivariable logistic regression analysis for each feature.

	OR	2.50%	97.50%	*P* value	OR	2.50 %	97.50%	*P* value	OR	2.50 %	97.50%	*P* value	OR	2.50 %	97.50%	*P* value
	Rosc on Scene				Survival to discharge from ED				30 day survival				cpc 1/2			
Location type																
Home residence	(Ref)															
Healthcare facility	1.21	0.96	1.52	0.11	1.49	1.26	1.75	< 0.05	1.91	1.51	2.40	< .05	1.91	1.35	2.63	< .05
Public/Commercial building	1.39	1.24	1.56	< .05	1.34	1.24	1.46	< 0.05	1.66	1.47	1.86	< .05	2.05	1.75	2.39	< .05
Nursing home	0.85	0.72	0.99	0.04	1.15	1.06	1.26	< 0.05	0.89	0.73	1.07	0.21	0.43	0.27	0.66	< .05
Street/highway	1.03	0.90	1.18	0.64	0.81	0.74	0.89	< 0.05	1.21	1.06	1.38	< .05	1.44	1.19	1.74	< .05
Industrial area	1.05	0.80	1.37	0.71	0.81	0.67	0.98	< .05	1.07	0.81	1.40	0.63	1.65	1.16	2.30	< .05
Transport center	1.20	0.77	1.80	0.39	0.99	0.71	1.35	0.94	0.94	0.56	1.50	0.80	0.99	0.48	1.86	0.99
Place of recreation	1.67	1.32	2.09	< .05	1.29	1.08	1.53	< .05	1.68	1.33	2.12	< .05	2.12	1.58	2.82	< .05
In EMS/Private ambulance	1.80	0.97	3.16	< .05	1.33	0.86	2.04	0.19	1.43	0.77	2.50	0.23	2.05	0.91	4.20	0.06
Other	1.13	0.98	1.31	0.09	1.08	0.99	1.18	0.08	1.36	1.19	1.56	< .05	1.44	1.19	1.73	< .05
Unknown	3.17	2.72	3.69	< 0.05	0.04	0.03	0.06	< .05	3.00	2.46	3.65	< .05	2.93	2.20	3.91	< .05
Gender																
Male																
Female	1.03	0.98	1.08	0.21	1.13	1.07	1.18	< .05	1.00	0.94	1.06	0.96	0.92	0.85	1.01	0.07
Age																
< 60																
60–69	0.97	0.91	1.04	0.40	0.95	0.89	1.01	0.10	0.85	0.80	0.91	< .05	0.76	0.69	0.83	< .05
70–79	0.91	0.86	0.97	< 0.05	0.85	0.80	0.90	< .05	0.67	0.63	0.72	< .05	0.59	0.53	0.65	< .05
> = 80	0.77	0.73	0.82	< 0.05	0.61	0.58	0.66	< .05	0.43	0.40	0.47	< .05	0.30	0.27	0.34	< .05
Arrest witnessed by																
Not witnessed																
EMS/Private ambulance	3.02	2.44	3.70	< 0.05	2.53	2.09	3.06	< .05	4.30	3.45	5.32	< .05	4.71	3.44	6.35	< .05
Bystander	2.53	2.41	2.65	< 0.05	2.01	1.92	2.11	< .05	2.69	2.53	2.85	< .05	2.62	2.39	2.87	< .05
Unknown	1.41	1.16	1.70	< 0.05	1.24	1.12	1.36	< .05	1.42	1.19	1.68	< .05	1.37	1.05	1.77	< .05
Bystander CPR																
No																
Yes	1.25	1.19	1.31	< 0.05	1.09	1.03	1.14	0.00	1.19	1.12	1.26	< .05	1.39	1.28	1.51	< .05
Unknown	1.44	0.72	2.62	0.27	1.10	0.77	1.55	0.58	1.18	0.58	2.17	0.63	0.55	0.09	1.86	0.42
First CPR initiated by																
No CPR initiated																
First responder	2.35	1.80	3.03	< 0.05	1.29	0.82	2.09	0.28	0.83	0.48	1.35	0.47	0.79	0.32	1.70	0.58
Ambulance crew	0.80	0.71	0.91	< 0.05	0.88	0.59	1.37	0.55	0.90	0.75	1.09	0.28	1.03	0.78	1.38	0.82
Bystander	0.95	0.84	1.08	0.46	0.85	0.57	1.33	0.46	1.13	0.94	1.37	0.19	1.27	0.96	1.69	0.09
Unknown	1.05	0.95	1.15	0.35	2.05	1.37	3.18	< .05	1.89	1.63	2.20	< .05	2.03	1.61	2.59	< .05
Bystander applied AED																
No																
Yes	1.28	1.11	1.47	< 0.05	1.14	0.95	1.37	0.16	1.38	1.18	1.61	< .05	1.38	1.14	1.67	< .05
Unknown	1.46	1.26	1.69	< 0.05	5.85	4.54	7.57	< .05	1.59	1.32	1.92	< .05	1.07	0.80	1.41	0.66
First arrest rhythm																
VF																
VT	1.41	1.11	1.79	< 0.05	0.73	0.56	0.95	0.02	0.81	0.62	1.04	0.11	0.85	0.61	1.17	0.35
PEA	0.61	0.56	0.67	< 0.05	0.66	0.60	0.74	< .05	0.49	0.44	0.54	< .05	0.40	0.35	0.46	< .05
Asystole	0.16	0.15	0.17	< 0.05	0.32	0.29	0.36	< .05	0.12	0.11	0.13	< .05	0.05	0.04	0.06	< .05
Unknown	0.87	0.79	0.97	< 0.05	0.52	0.47	0.58	< .05	0.66	0.59	0.74	< .05	0.94	0.82	1.09	0.43
Prehospital defibrillation																
No																
Yes	1.38	1.23	1.54	< 0.05	0.53	0.42	0.67	< .05	1.45	1.26	1.66	0.00	2.04	1.66	2.50	0.00
Unknown					1.16	0.06	8.19	0.90	2.51	0.12	18.53	0.42				
Defibrillation performed by first responder																
No																
Yes	1.05	0.86	1.29	0.62	0.67	0.07	6.40	0.78	1.45	1.16	1.81	0.00	1.21	0.90	1.61	0.20
Unknown	1.24	0.82	1.92	0.32	0.45	0.05	4.40	0.56	0.73	0.46	1.19	0.19	0.73	0.42	1.31	0.27
Defibrillation performed by ambulance crew																
No																
Yes	1.10	0.97	1.25	0.15	2.21	1.73	2.84	0.00	1.41	1.22	1.65	0.00	1.60	1.28	2.00	0.00
Unknown	1.30	1.18	1.44	0.00	0.94	0.87	1.02	0.17	1.26	1.11	1.42	0.00	1.43	1.18	1.74	0.00
Defibrillation performed by bystander-lay person																
No																
Yes	0.99	0.60	1.61	0.97	12.02	1.20	118.29	0.07	1.53	0.89	2.58	0.12	1.66	0.87	3.05	0.11
Unknown	0.69	0.44	1.05	0.09	8.56	0.88	86.16	0.12	1.86	1.14	2.95	0.01	1.81	0.99	3.18	0.05
Mechanical CPR device used by EMS/private ambulance																
No																
Yes	0.63	0.55	0.72	0.00	0.93	0.84	1.03	0.19	0.64	0.53	0.76	0.00	0.98	0.75	1.26	0.87
Unknown	0.99	0.91	1.07	0.72	0.23	0.18	0.28	0.00	0.95	0.87	1.05	0.34	1.09	0.94	1.27	0.24
Prehospital advanced airway																
No																
Yes	0.81	0.77	0.84	0.00	1.11	1.06	1.17	0.00	0.77	0.73	0.82	0.00	0.52	0.48	0.56	0.00
Unknown	0.63	0.44	0.87	0.01	1.22	1.05	1.42	0.01	1.26	0.99	1.59	0.06	0.59	0.38	0.89	0.02
Prehospital epinephrine administered																
No																
Yes	3.96	3.76	4.17	0.00	1.77	1.62	1.94	0.00	0.88	0.81	0.95	0.00	0.43	0.38	0.48	0.00
Unknown	1.58	1.38	1.80	0.00	1.50	1.32	1.70	0.00	1.47	1.23	1.76	0.00	0.90	0.68	1.17	0.43

ROSC, return of spontaneous circulation; EMS, emergency medical service; CPR, cardiopulmonary resuscitation; AED, automated external defibrillator; VT, ventricular tachycardia; VF, ventricular fibrillation; PEA, pulseless electrical activity; ET, endotracheal intubation.

## Discussion

PReCAP is a model that can predict OHCA in real-time during prehospital care. Through the development of a time-adaptive cohort and each time-adaptive cohort-based model, we were able to predict ROSC on scene with an AUROC score greater than 0.8. The potential for ROSC at the scene is crucial for patient outcomes^3,8^. PReCAP study also includes further long-term predictions, including outcomes of ED, 30-day survival, and CPC. PReCAP also had high AUROC scores (more than 0.8) for most minutes. By providing prediction of possible ROSC at the scene and long-term outcomes, this model can support prehospital decisions, such as when to initiate the transfer, and where to transfer.

To the best of our knowledge, PReCAP is the real-time prediction in the prehospital phase. Few machine-learning-based models make predictions in the prehospital phase. The majority of machine-learning-based prediction models for OHCA are made using in-hospital variables and are developed for hospital settings^20,21,24–26^. In addition, only a few of them make predictions in real-time at the prehospital stage of OHCA.

Each time cohort has a different prediction model, and the feature importance was also different among time-adaptive cohorts (Figs. S3–S5 Multimedia Appendix 1). This might refer to the effect of factors that affect the changes in patient prognosis during CPR.

Each variable’s specific relevance to scene ROSC is not explainable in our study. For example, advanced airway shows a negative effect on scene ROSC in multivariable analysis. However, there might be some bias related to the performer’s skill level or situational factors that a retrospective study cannot consider. If chest compression was interrupted or defibrillation was delayed due to performing advanced airway skills, it might result in a bad outcome on the scene. For example, epinephrine injection shows high feature importance in the initial stage of EMS CPR; however, feature importance itself decreases as time goes on. In multivariable analysis, epinephrine injection shows relevance with scene ROSC in our study. However, this study is a retrospective study, and the non-strictly controlled effect of epinephrine injection in the prehospital stage could be discussed in further study.

PReCAP employs a time-adaptive cohort with distinct prediction results for every minute. Consequently, new predictions are made every minute. This may assist the EMS team in making real-time decisions at the arrest scene. In addition, as seen in a previous study, the time-adaptive cohort-based model can predict arrest outcomes similar to the real world^24^. Our model also shows a similar trend in the real world. As time goes on, the survival rate decreases sharply in our prediction model, which correlates with real-world results (Fig. 2, Table S6 of Multimedia Appendix 1)^4,24,27^. This is because it excludes patients for each minute and re-predicts the results based on time. This is one aspect wherein PReCAP reflects the real world, as it’s prediction result changes as times changes.

PReCAP is based on the multinational database PAROS, which includes prehospital information of OHCA patient from thirteen countries^28^. Consequently, this database contains populations with varying characteristics. By utilizing this database, we anticipate that the performance of this model will be comparable to that of the development database, even in the most diverse populations. However, country, and city details were omitted from the prediction input variable when using this database. We omitted these variables to apply this model to countries and cities not included in the PAROS database, hope that this model could be validated in another environment. On the other hand, given that local elements like the EMS system and rules can have an impact on the decision, country and city data may be helpful when applying this approach in the actual world. For each country or region to use this model, it may be necessary to create multiple versions in order to further customize it for use in the real world.

PReCAP makes use of prehospital obtainable variables; thus, it is applicable to real-world settings. Because it is difficult to obtain information regarding presumed causes of cardiac arrest and hospital treatment during the prehospital phase, it is not applied. In the real world, past medical history cannot be precisely defined during the prehospital phase, especially if the arrest occurs in the absence of witnesses. We selected input variables based on the clarity of their definitions, which can be obtained at the scene during the prehospital phase.

However, we did not perform the exact implementation of this model. There are some hurdles to doing this, like how these variables might be entered for prediction in a real-scene environment. Additional interface systems, such as dashboards or application development, are needed for real-world implementation. Further study is needed for the implementation of this model.

If it is possible to apply this model in the real world, after further development, this model might be helpful for decision-making during the prehospital phase, such as transport time and which hospital to transport, by providing the prediction results of scene outcome and long-term outcome.

### Limitation

There are several limitations to this study. First, this is a retrospective observational study. Therefore, there might be a selection bias and the model might show decreased performance in different populations. However, by including a large population, we tried to create a model that could be widely applied in a diverse environment. Second, we did not conduct external validation. However, the database used for this model development includes a multinational population in Asia. Therefore, this model was first trained and tested with multinational data. It already includes a variety of populations and environments in model training, which might minimize the limitation that the performance of the prediction model could decrease in an external environment. However, further study is required to prove its performance in other environments or populations. Thirdly, as mentioned above, the real-world implications are not covered by this study. For real-world use, a system for entering data, calculating result, and an interface should also be developed. This is needed for further study. Fourthly, data was reshuffled each minute as the dataset size changed in each progressive time-adaptive cohort. By making separate cohort by each minute, authors made independently separate prediction model for each time cohort, to avoid problem of using same patient in next model.

## Conclusion

PReCAP had an AUROC score between 0.8 and 0.95 for predicting ROSC on scene, survival to discharge from ED, 30-day survival, and CPC by each minute, using the PAROS database. As this model used a multinational database, it may be applicable for a variety of environments and populations. By using a time-adaptive model and predicting outcomes by each minute, this model might be beneficial for real-time decision-making in a prehospital environment after additional further study for practical implementation.

### Supplementary Information

Supplementary Information.

## Data Availability

Data was obtained from the Pan-Asian Resuscitation Outcome Study (PAROS). The datasets generated and analyzed during the current study are not publicly available because although the dataset is de-identified it includes some patient information. However, the datasets are available from PAROS on reasonable request. (Contact: patricia.tay@scri.cris.sg.).

## Abbreviations

OHCA: Out-of-hospital cardiac arrest
ROSC: Return of spontaneous circulation
ACLS: Advanced cardiac life support
EMS: Emergency medical service
PReCAP: Pre-hospital Real-time Cardiac Arrest outcome Prediction
IRB: Institutional Review Board
PAROS: Pan-Asian Resuscitation Outcome Study
ED: Emergency department
CPC: Cerebral Performance Category
CPR: Cardiopulmonary resuscitation
AED: Automated external defibrillator
AUROC: Area under the receiver operating characteristic curve
VT: Ventricular tachycardia
VF: Ventricular fibrillation
PEA: Pulseless electrical activity
ET: Endotracheal intubation

## References

[1] Chang H, Jeong D, Park JE (2022). Prehospital airway management for out-of-hospital cardiac arrest: A nationwide multicenter study from the KoCARC registry. Acad. Emerg. Med..

[2] Lee SK, Kim GW, Kim CH (2014). Prehospital cardiopulmonary resuscitation by 119 emergency medical technician (EMT) for increasing the rate of return of spontaneous circulation; national-wide 119 EMT survey. J. Korean Soc. Emerg. Med..

[3] Ong MEH, Perkins GD, Cariou A (2018). Out-of-hospital cardiac arrest: Prehospital management. Lancet.

[4] Panchal AR, Bartos JA, Cabanas JG (2020). Part 3: Adult basic and advanced life support: 2020 American heart association guidelines for cardiopulmonary resuscitation and emergency cardiovascular care. Circulation.

[5] Perkins GD, Olasveengen TM, Maconochie I (2018). European resuscitation council guidelines for resuscitation: 2017 update. Resuscitation.

[6] Jang Y, Kim TH, Lee SY (2022). Association of transport time interval with neurologic outcome in out-of-hospital cardiac arrest patients without return of spontaneous circulation on scene and the interaction effect according to prehospital airway management. Clin. Exp. Emerg. Med..

[7] Lo AX (2020). Challenging the "Scoop and Run" model for management of out-of-hospital cardiac arrest. JAMA.

[8] Smith RM, Conn AK (2009). Prehospital care—scoop and run or stay and play?. Injury.

[9] Dick WF (2003). Anglo-American vs. Franco-German emergency medical services system. Prehosp. Disaster Med..

[10] Grunau B, Kime N, Leroux B (2020). Association of intra-arrest transport vs continued on-scene resuscitation with survival to hospital discharge among patients with out-of-hospital cardiac arrest. JAMA.

[11] Krarup NH, Terkelsen CJ, Johnsen SP (2011). Quality of cardiopulmonary resuscitation in out-of-hospital cardiac arrest is hampered by interruptions in chest compressions—a nationwide prospective feasibility study. Resuscitation.

[12] Cheskes S, Byers A, Zhan C (2017). CPR quality during out-of-hospital cardiac arrest transport. Resuscitation.

[13] Cha K-C, Hwang S-O (2023). The future of resuscitation. Clin. Exp. Emerg. Med..

[14] Zive D, Koprowicz K, Schmidt T (2011). Variation in out-of-hospital cardiac arrest resuscitation and transport practices in the Resuscitation Outcomes Consortium: ROC Epistry-Cardiac Arrest. Resuscitation.

[15] Nichol G, Thomas E, Callaway CW (2008). Regional variation in out-of-hospital cardiac arrest incidence and outcome. JAMA.

[16] Baldi E, Caputo ML, Savastano S (2020). An Utstein-based model score to predict survival to hospital admission: The UB-ROSC score. Int. J. Cardiol..

[17] Coult J, Yang BY, Kwok H (2023). Prediction of shock-refractory ventricular fibrillation during resuscitation of out-of-hospital cardiac arrest. Circulation.

[18] Lupton JR, Jui J, Neth MR, Sahni R, Daya MR, Newgard CD (2022). Development of a clinical decision rule for the early prediction of shock-refractory out-of-hospital cardiac arrest. Resuscitation.

[19] Navab E, Esmaeili M, Poorkhorshidi N, Salimi R, Khazaei A, Moghimbeigi A (2019). Predictors of out of hospital cardiac arrest outcomes in pre-hospital settings; a retrospective cross-sectional study. Arch. Acad. Emerg. Med..

[20] Park JH, Choi J, Lee S, Shin SD, Song KJ (2022). Use of time-to-event analysis to develop on-scene return of spontaneous circulation prediction for out-of-hospital cardiac arrest patients. Ann. Emerg. Med..

[21] Liu N, Liu M, Chen X (2022). Development and validation of an interpretable prehospital return of spontaneous circulation (P-ROSC) score for patients with out-of-hospital cardiac arrest using machine learning: A retrospective study. EClinicalMedicine.

[22] Virani SS, Alonso A, Benjamin EJ (2020). Heart disease and stroke statistics-2020 update: A report from the American Heart Association. Circulation.

[23] Ke G, Meng Q, Finley T (2017). Lightgbm: A highly efficient gradient boosting decision tree. Adv. Neural Inf. Process. Syst..

[24] Kim JW, Ha J, Kim T (2021). Developing a time-adaptive prediction model for out-of-hospital cardiac arrest: Nationwide cohort study in Korea. J. Med. Internet Res..

[25] Lee Y, Kwon JM, Lee Y, Park H, Cho H, Park J (2018). Deep learning in the medical domain: Predicting cardiac arrest using deep learning. Acute Crit. Care.

[26] Seki T, Tamura T, Suzuki M, Group S-KS (2019). Outcome prediction of out-of-hospital cardiac arrest with presumed cardiac aetiology using an advanced machine learning technique. Resuscitation.

[27] Link MS, Berkow LC, Kudenchuk PJ (2015). Part 7: Adult advanced cardiovascular life support: 2015 American Heart Association Guidelines update for cardiopulmonary resuscitation and emergency cardiovascular care. Circulation.

[28] Doctor NE, Ahmad NSB, Pek PP, Yap S, Ong MEH (2017). The Pan-Asian Resuscitation Outcomes Study (PAROS) clinical research network: what, Where, why and how. Singap. Med. J..

