# Supplementary material for: Machine learning pre-hospital real-time cardiac arrest outcome prediction (PReCAP) using time-adaptive cohort model based on the Pan-Asian Resuscitation Outcome Study
Source: Sci Rep. 2023 Nov 21;13:20344. doi: 10.1038/s41598-023-45767-z (PMC10663550; doi:10.1038/s41598-023-45767-z)
Supplement: Supplementary file 1 — Supplementary Information. [file 41598_2023_45767_MOESM1_ESM.pdf]

Supplementary Table S1. Predictors Used for PReCAP

**Location type**	
	Home residence
	Healthcare facility
	Public/Commercial building
	Nursing home
	Street/highway
	Industrial area
	Transport center
	Place of recreation
	In EMS/Private ambulance
	Other
	Unknown
**Gender**	
	Male
	Female
**Age**	
	< 60
	60–69
	70–79
	>= 80
**Arrest witnessed by**	
	Not witnessed
	EMS/Private ambulance
	Bystander
	Unknown
**Bystander CPR**	
	No
	Yes
	Unknown
**First CPR initiated by**	
	No CPR initiated
	First responder
	Ambulance crew
	Bystander
	Unknown
**Bystander applied AED**	
	No

	Yes
	Unknown
**First arrest rhythm**	
	VF
	VT
	PEA
	Asystole
	Unknown
**Prehospital defibrillation**	
	No
	Yes
	Unknown
**Defibrillation performed by first responder**	
	No
	Yes
	Unknown
**Defibrillation performed by ambulance crew**	
	No
	Yes
	Unknown
**Defibrillation performed by bystander-lay person**	
	No
	Yes
	Unknown
**Mechanical CPR device used by EMS/private ambulance**	
	No
	Yes
	Unknown
**Prehospital advanced airway**	
	No
	Yes
	Unknown
**Prehospital epinephrine administered**	
	No
	Yes
	Unknown

Supplementary Table S2. Population of each country included in the PAROS data.

Country	n (%)
**China**	767 (0.5)
**India**	18 (0.0)
**Indonesia**	1 (0.0)
**Japan**	119,316 (75.7)
**Korea**	18,631 (11.8)
**Lebanon**	8 (0.0)
**Malaysia**	729 (0.5)
**Philippines**	94 (0.1)
**Singapore**	10,661 (6.8)
**Taiwan**	5807 (3.7)
**Thailand**	508 (0.3)
**UAE**	1100 (0.7)
**Vietnam**	14 (0.0)

Supplementary Table S3. The prediction of ROSC on scene by Pre-Hospital Real Time Precision Model (PReCAP).

	Accuracy	Precision	Recall	Specificity	AUROC
**0 min**	0.926	0.620	0.076	0.996	0.864
**1 min**	0.926	0.593	0.068	0.996	0.864
**2 min**	0.928	0.614	0.053	0.997	0.864
**3 min**	0.931	0.572	0.038	0.998	0.863
**4 min**	0.934	0.652	0.015	0.999	0.862
**5 min**	0.938	0.571	0.008	0.999	0.862
**6 min**	0.941	0.667	0.006	0.999	0.861
**7 min**	0.945	0.500	0.002	0.999	0.860
**8 min**	0.947	0.625	0.003	0.999	0.862
**9 min**	0.950	0.600	0.004	0.999	0.862
**10 min**	0.954	0.714	0.004	0.999	0.866
**11 min**	0.957	0.500	0.001	0.999	0.867
**12 min**	0.960	0.500	0.003	0.999	0.868
**13 min**	0.962	0.500	0.001	0.999	0.870
**14 min**	0.964	0.500	0.001	0.999	0.867
**15 min**	0.966	0.600	0.004	0.999	0.872
**16 min**	0.968	0.667	0.003	0.999	0.870
**17 min**	0.970	0.333	0.002	0.999	0.868
**18 min**	0.972	0.500	0.004	0.999	0.866
**19 min**	0.973	0.500	0.004	0.999	0.868
**20 min**	0.974	0.001	0.001	0.999	0.867

Supplementary Table S4. The prediction of Emergency Department survival Discharge by Pre-Hospital Real Time Precision Model (PReCAP)**.**

	**Accuracy**	**Precision**	**Recall**	**Specificity**	**AUROC**
**0 min**	0.929	0.615	0.128	0.993	0.918
**1 min**	0.928	0.609	0.127	0.993	0.919
**2 min**	0.929	0.603	0.123	0.993	0.918
**3 min**	0.930	0.607	0.122	0.994	0.918
**4 min**	0.930	0.585	0.113	0.994	0.917
**5 min**	0.931	0.578	0.107	0.994	0.916
**6 min**	0.932	0.570	0.082	0.995	0.916
**7 min**	0.933	0.567	0.070	0.996	0.917
**8 min**	0.935	0.583	0.061	0.997	0.918
**9 min**	0.937	0.547	0.043	0.998	0.918
**10 min**	0.939	0.590	0.041	0.998	0.920
**11 min**	0.941	0.565	0.021	0.999	0.921
**12 min**	0.943	0.419	0.012	0.999	0.923
**13 min**	0.946	0.394	0.009	0.999	0.924
**14 min**	0.948	0.333	0.008	0.999	0.924
**15 min**	0.950	0.292	0.006	0.999	0.926
**16 min**	0.953	0.412	0.007	0.999	0.929
**17 min**	0.956	0.353	0.007	0.999	0.929
**18 min**	0.957	0.361	0.016	0.999	0.930
**19 min**	0.959	0.282	0.016	0.998	0.931
**20 min**	0.961	0.400	0.013	0.999	0.932

Supplementary Table S5. The prediction of 30-day survival by Pre-Hospital Real Time Precision Model (PReCAP)**.**

	**Accuracy**	**Precision**	**Recall**	**Specificty**	**AUROC**
**0 min**	0.953	0.606	0.112	0.112	0.852
**1 min**	0.953	0.593	0.094	0.094	0.851
**2 min**	0.955	0.616	0.101	0.101	0.848
**3 min**	0.956	0.594	0.081	0.081	0.846
**4 min**	0.958	0.556	0.057	0.057	0.842
**5 min**	0.960	0.481	0.030	0.030	0.840
**6 min**	0.962	0.370	0.009	0.009	0.835
**7 min**	0.964	0.379	0.010	0.010	0.833
**8 min**	0.967	0.400	0.004	0.004	0.829
**9 min**	0.969	0.333	0.003	0.003	0.824
**10 min**	0.971	0.429	0.004	0.004	0.821
**11 min**	0.973	0.200	0.001	0.001	0.822
**12 min**	0.975	0.001	0.001	0.001	0.824
**13 min**	0.977	0.001	0.001	0.001	0.821
**14 min**	0.979	0.001	0.001	0.001	0.817
**15 min**	0.980	0.001	0.001	0.001	0.819
**16 min**	0.981	0.001	0.001	0.001	0.811
**17 min**	0.982	0.400	0.006	0.006	0.807
**18 min**	0.984	0.500	0.006	0.006	0.802
**19 min**	0.985	0.500	0.004	0.004	0.805
**20 min**	0.986	0.001	0.001	0.000	0.803

Supplementary Table S6. The prediction of Cerebral Performance Category 1 or 2 by Pre-Hospital Real Time Precision Model (PReCAP)**.**

	**Accuracy**	**Precision**	**Recall**	**AUROC**
**0 min**	0.976	0.479	0.122	0.917
**1 min**	0.977	0.480	0.119	0.915
**2 min**	0.978	0.455	0.103	0.915
**3 min**	0.980	0.480	0.097	0.914
**4 min**	0.981	0.440	0.069	0.911
**5 min**	0.983	0.417	0.058	0.908
**6 min**	0.985	0.393	0.024	0.902
**7 min**	0.986	0.346	0.022	0.902
**8 min**	0.987	0.250	0.022	0.899
**9 min**	0.989	0.500	0.003	0.892
**10 min**	0.990	0.167	0.007	0.887
**11 min**	0.991	0.091	0.004	0.882
**12 min**	0.992	0.333	0.005	0.871
**13 min**	0.992	0.001	0.001	0.871
**14 min**	0.993	0.001	0.001	0.869
**15 min**	0.993	0.001	0.001	0.864
**16 min**	0.993	0.001	0.001	0.866
**17 min**	0.994	0.300	0.025	0.870
**18 min**	0.995	0.400	0.021	0.864
**19 min**	0.995	0.250	0.024	0.835
**20 min**	0.995	0.333	0.013	0.840

Supplementary Table S7. The predicted survival rate by time in Pre-Hospital Real Time Precision Model (PReCAP), conventional model, and real-world ROSC on scene rate**.**

	Conventional model	PReCAP	Real world
**1 min**	0.077047	0.077047	0.076
**2 min**	0.076708	0.074496	0.075
**3 min**	0.076319	0.07256	0.073
**4 min**	0.075605	0.068641	0.069
**5 min**	0.074802	0.065282	0.066
**6 min**	0.073948	0.061321	0.062
**7 min**	0.073313	0.057638	0.059
**8 min**	0.072704	0.053837	0.055
**9 min**	0.072158	0.050969	0.052
**10 min**	0.071585	0.048002	0.049
**11 min**	0.071238	0.045141	0.046
**12 min**	0.070992	0.041908	0.043
**13 min**	0.070959	0.039441	0.040
**14 min**	0.070991	0.037222	0.038
**15 min**	0.071121	0.03483	0.036
**16 min**	0.07139	0.032791	0.034
**17 min**	0.071467	0.030903	0.032
**18 min**	0.072129	0.0294	0.030
**19 min**	0.072352	0.027347	0.028
**20 min**	0.073034	0.026236	0.076

PReCAP=Pre-hospital Real-time Cardiac Arrest Prediction.

Supplementary Figure S1. Feature importance of Emergency Department survival Discharge in Pre-Hospital Real Time Precision Model (PReCAP) at 0 min , 5min and 10min.

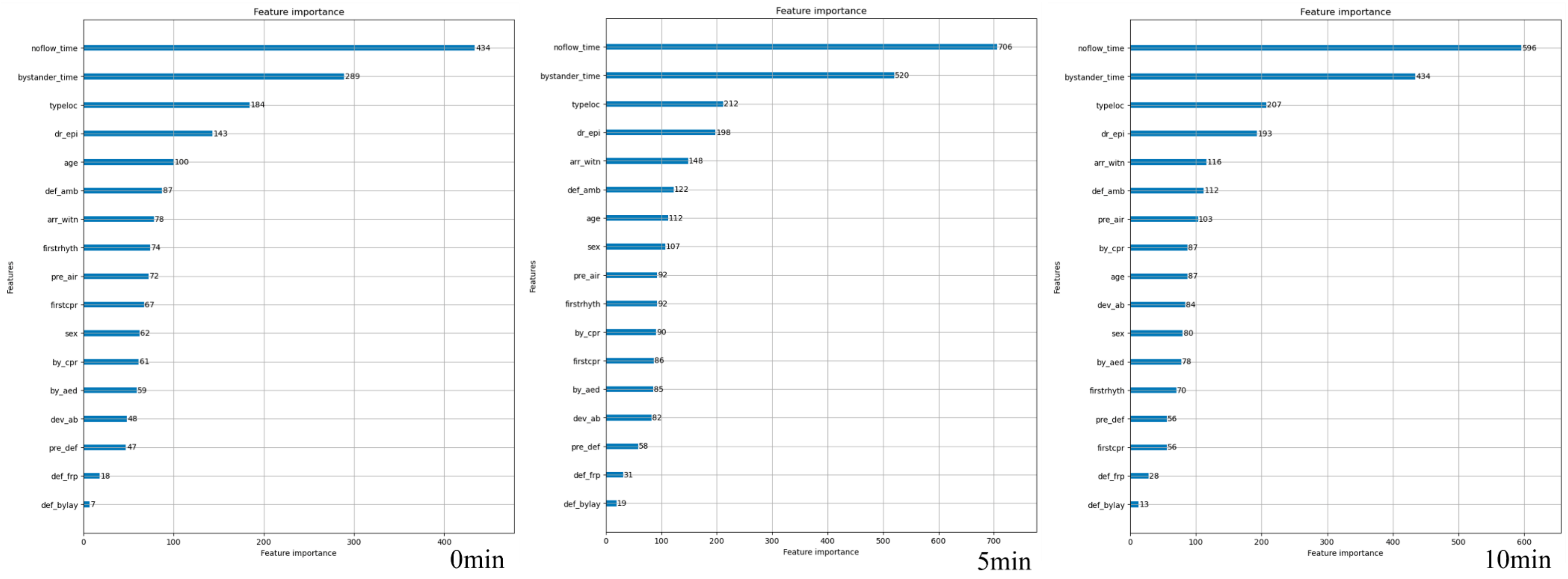

noflow_time: Estimated Occurrence time to response time, bystander_time: Bystander CPR time, dr_epi: Prehospital Epinephrine injection, firstrhyth: First Rhythm, typeloc: Location type, pre_air: Prehospital advanced airway, arr_witn: Witnessed arrest, by_aed: Bystander AED done, def_amb: Defibrillation in ambulance, frstcpr: First CPR initiator, by_cpr: Bystander CPR done, dev_ab: Mechanical CPR device used by EMS/private ambulance, pre_def: Prehospital defibrillation, def_frp: Defibrilation performed by first responder, def_bylay: Defibrillation performed by bystander-lay person

Supplementary Figure S2. Feature importance of 30-day survival by in Pre-Hospital Real Time Precision Model (PReCAP) at 0 min , 5min and 10min.

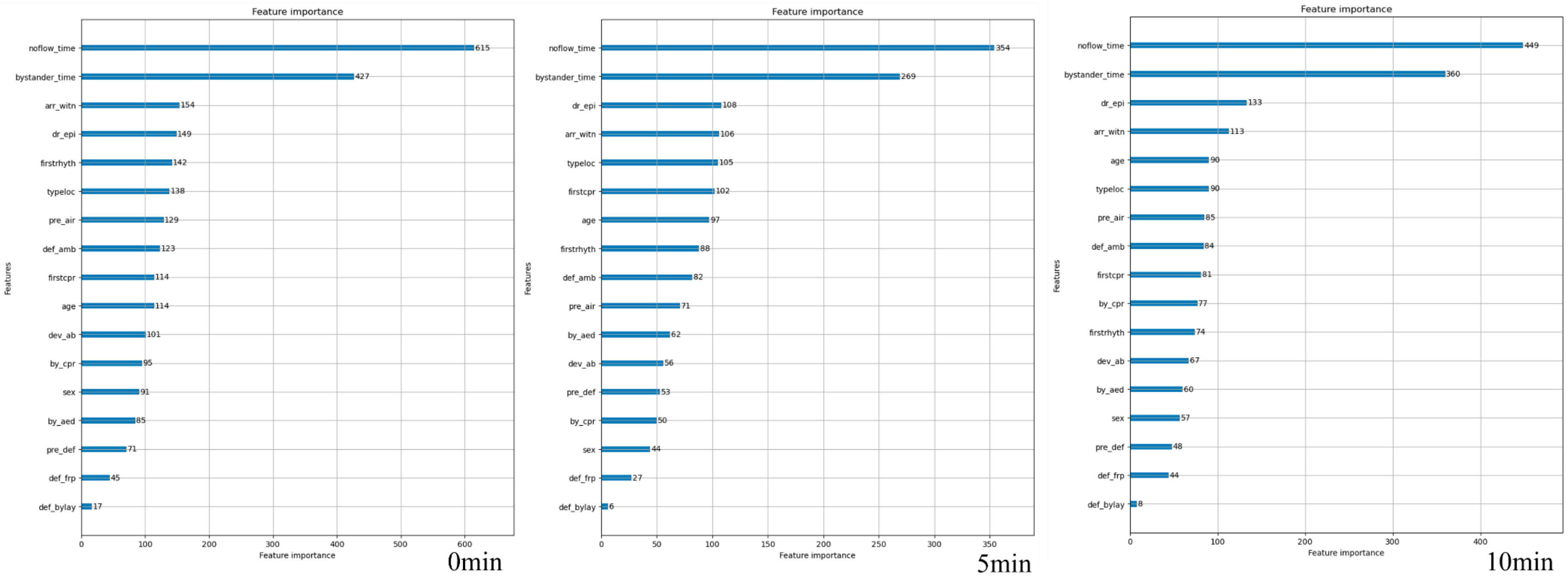

noflow_time: Estimated Occurrence time to response time, bystander_time: Bystander CPR time, dr_epi: Prehospital Epinephrine injection, firstrhyth: First Rhythm, typeloc: Location type, pre_air: Prehospital advanced airway, arr_witn: Witnessed arrest, by_aed: Bystander AED done, def_amb: Defibrillation in ambulance, frstcpr: First CPR initiator, by_cpr: Bystander CPR done, dev_ab: Mechanical CPR device used by EMS/private ambulance, pre_def: Prehospital defibrillation, def_frp: Defibrilation performed by first responder, def_bylay: Defibrillation performed by bystander-lay person

Supplementary Figure S3. Feature importance of Cerebral Performance Category 1 and 2 in Pre-Hospital Real Time Precision Model (PReCAP) 0 min , 5min and 10min.

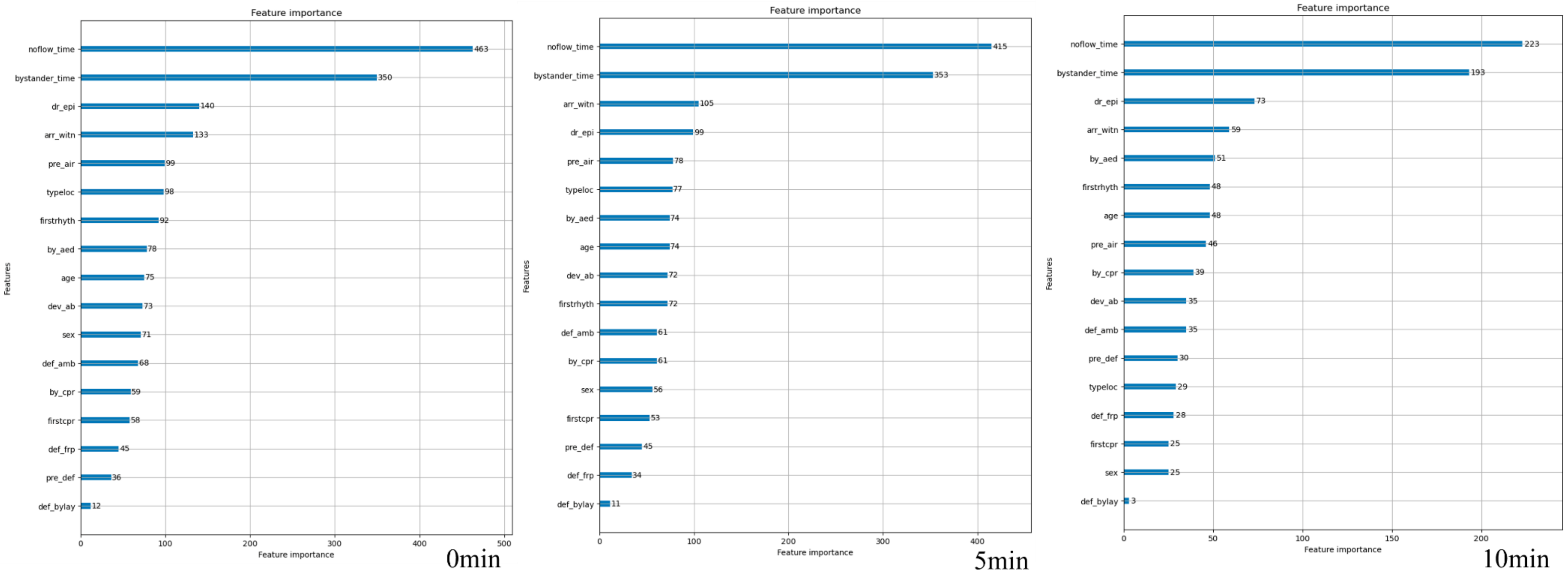

noflow_time: Estimated Occurrence time to response time, bystander_time: Bystander CPR time, dr_epi: Prehospital Epinephrine injection, firstrhyth: First Rhythm, typeloc: Location type, pre_air: Prehospital advanced airway, arr_witn: Witnessed arrest, by_aed: Bystander AED done, def_amb: Defibrillation in ambulance, frstcpr: First CPR initiator, by_cpr: Bystander CPR done, dev_ab: Mechanical CPR device used by EMS/private ambulance, pre_def: Prehospital defibrillation, def_frp: Defibrilation performed by first responder, def_bylay: Defibrillation performed by bystander-lay person.
